# Model Development of a Novel Robotic Surgery Training Exercise With Electrocautery

**DOI:** 10.7759/cureus.24531

**Published:** 2022-04-27

**Authors:** Christina S Lee, Mustafa T Khan, Ronit Patnaik, Mamie C Stull, Robert W Krell, Robert B Laverty

**Affiliations:** 1 General Surgery, Brooke Army Medical Center, San Antonio, USA; 2 General Surgery, UT (University of Texas ) Health San Antonio, San Antonio, USA

**Keywords:** tool development, tissue model, low-cost high-fidelity task trainers, electrocautery training, inanimate model, skills and simulation training, robotic assited surgery

## Abstract

Robot-assisted surgery (RAS) has undergone rapid adoption in general surgery due to features such as three-dimensional visualization, wrist dexterity, improved precision of movement, and operator ergonomics. While many surgical trainees encounter RAS during their residency, robotic skills training programs and curricula vary across institutions and there is broad variation in graduating general surgeons’ robotic proficiency levels. Due to a need for a formalized process to achieve competence on the robotic platform, simulation-based training has become instrumental in closing this gap as it provides training in a low-stakes environment while allowing the trainee to improve their psychomotor and basic procedural skills.

Several different models of simulation training exist including virtual reality, animal, cadaveric, and inanimate tissue platforms. Each form of training has its own merits and limitations. While virtual reality platforms have been well evaluated for face, content, and construct validity, their initial set-up costs can be as high as $125,000. Similarly, animal and cadaveric models are not only costly but also have ethical considerations that may preclude participation. There is an unmet need in developing high-fidelity, cost-effective simulations for basic videoscopic skills such as cautery use.

We developed a cost-effective and high-fidelity inanimate tissue model that incorporates electrocautery. Using a double-layered bowel model secured to a moistened household sponge, this inanimate exercise simulates fundamental skills of robotic surgery such as tissue handling, camera control, suturing, and electrocautery.

## Introduction

The improved dexterity, precision, three-dimensional visualization, and ergonomics that are inherent to robot-assisted surgery (RAS) have allowed it to rapidly transition from a novel platform to a widely used tool that is quickly becoming incorporated into surgical practice. Despite the rapid uptake of the technology, there remains a lack of standardized training and evaluation platforms for robotic proficiency [1]. This has led to increased interest in developing valid and cost-effective training mechanisms in robotic surgery.

In 2006, the Society of American Gastrointestinal and Endoscopic Surgeons (SAGES) and Minimally Invasive Robotic Association (MIRA) published a document suggesting that the ideal RAS curriculum should consist of didactics, hands-on training, and mentored surgical experiences [2]. Due to limited resident exposure to RAS during general surgery residency, simulation has become an important aspect of training. Simulation allows the trainee to perform key components of a procedure in a low-stakes environment while improving their psychomotor and basic procedural skills. Such training is well established in laparoscopic surgery and has become mandatory for board certification and hospital credentialing in the United States (US); but no such requirements exist for RAS [3,4]. Virtual reality trainers are among the most validated in the realm of robotic simulation training; however, they can be prohibitively expensive and are known to poorly emulate electrocautery [5,6].

Virtual simulation of electrosurgery has been the subject of physical science research since the introduction of laparoscopic surgery. Due to the different conducting activities of muscles, fat, fascia, etc., in the body, the thermal spread of the electrocautery is difficult to measure and predict [7]. There exist multiple simulation models for electrosurgery in the laparoscopic platform using both monopolar and bipolar energy sources, yet these are cost-prohibitive and there have not been adaptations to the robotic platform based on our most current search [8,9]. Similarly, the use of electrocautery in inanimate exercises is lacking in published literature.

In this technical report, we demonstrate a cost-effective and high-fidelity inanimate tissue model that effectively incorporates electrocautery and is relevant to surgical training. This model can easily be adapted to any RAS training curriculum for teaching and testing the fundamentals of robotic surgery to include electrocautery, tissue handling, camera control, and suturing.

## Technical report

Model set-up

The materials collected for the creation of the inanimate exercise are listed in Table 1.

**Table 1:** Itemized list of materials used in the exercise. (*) marks single-use materials; (±) indicates items that can be obtained and reused through a hospital’s SPD; the cost of the materials represents the best estimate of market price per unit in USD ($). SPD: sterile processing department Intuitive Surgical, Inc., Sunnyvale, California, United States; LifeLike BioTissue, London, Ontario, Canada; Ethicon, Inc., Raritan, New Jersey, United States (Vicryl suture)

Item	Cost (per unit)
Heavy duty cleaning sponge*	$0.86
Marking pen*	$1.70
Electrocautery grounding pad*	$146.90
Needle driver^±^	$975.50
Suture scissors^±^	$37.48
Intuitive Abdominal Dome Trainer	$1000
Intuitive Cadiere/bipolar/ProGrasp forceps^±^	$1500
Intuitive Monopolar scissors^±^	$1500
Intuitive Mega Suture Cut^TM^ Needle Driver^±^	$1878.24
Intuitive Zero-degree endoscopic camera^±^	$625
Monopolar cord^±^	$51.50
LifeLike Bowel model*	$42.50
3-0 Vicryl suture*	$4.40
Saline flush*	$0.22
Silk tape*	$2.28
Total Set up (without use of SPD)	$7,766.58
Total Set up (with use of SPD)	$1,198.86
Total per use (with use of SPD)	$198.86

**Figure 1:**
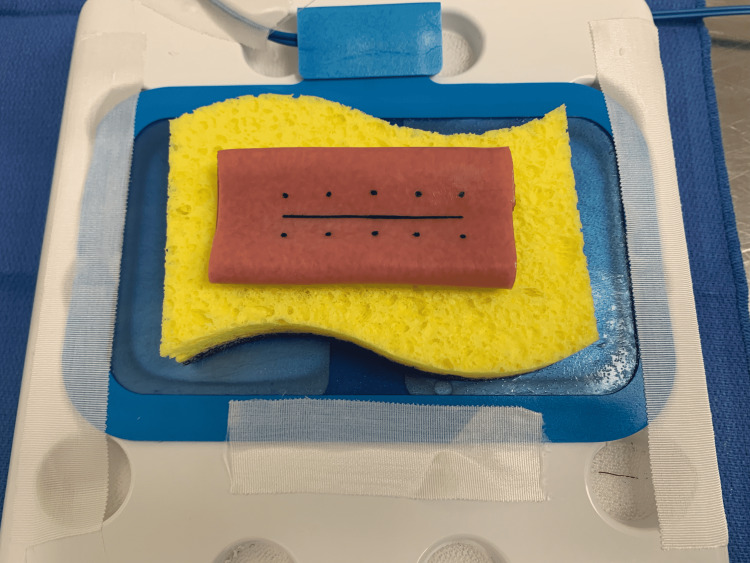
Inanimate training exercise set-up with heavy-duty cleaning sponge on an electrocautery grounding pad secured to the LifeLike Biotissue double layered bowel model. LifeLike Biotissue, London, Ontario, Canada

The costs for each of these items are also listed, which were according to contracted agreements with manufacturers and the Department of Defense. The base of the bowel model was constructed using a six-inch double-layered bowel model (LifeLike BioTissue Inc., London, Ontario, Canada). This was secured to a heavy-duty cleaning sponge (stripped of its scratchpad) with interrupted 3-0 silk sutures at the four corners of the bowel model. The bowel was then marked with a longitudinal incision line measuring 5cm with five pairs of dots evenly spaced 1cm from each other and 5mm from the incision line to mark locations for the suture needle to enter and exit the bowel. Both sides of the bowel were marked in this manner. This was done with a plastic stencil, which was laser engraved to ensure consistency amongst the models. We then secured an electrocautery grounding pad to the base of the robot abdominal dome trainer with silk medical tape. Following this, the sponge was moistened with 5cc of normal saline and the sponge-bowel model was attached to the adhesive portion of the electrocautery grounding pad (Figure 1). 

Robot set-up

**Figure 2:**
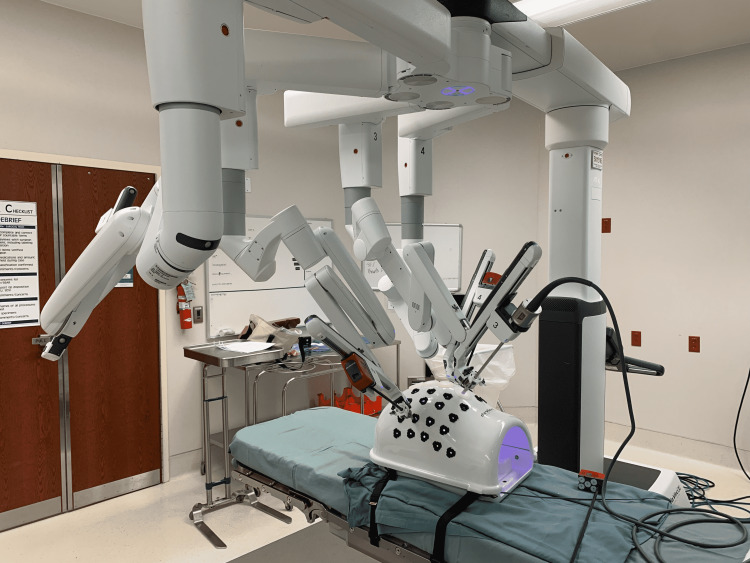
da Vinci arm set-up in the Intuitive abdominal dome model: Arm 1 is stowed; the camera was placed on the center arm (Arm 3) with grasper on the left hand (Arm 2) and either monopolar scissors or needle driver on the right hand (Arm 4). Intuitive Surgical, Inc., Sunnyvale, California, United States

The da Vinci Xi (Intuitive Surgical, Inc., Sunnyvale, California, United States) console was used during the exercises. Arm 1 was stowed. The camera was placed into Arm 3 in the center of the abdominal dome model (Figure 2). Forceps (Cadiere, bipolar fenestrated, or ProGrasp are acceptable) were placed into Arm 2 and monopolar scissors were placed into Arm 4 during the enterotomy portion, which was then switched to the Mega Suture Cut^TM^ needle driver (Intuitive Surgical, Inc., Sunnyvale, California, United States) during the suturing portion of the model. 

Training exercises

**Figure 3:**
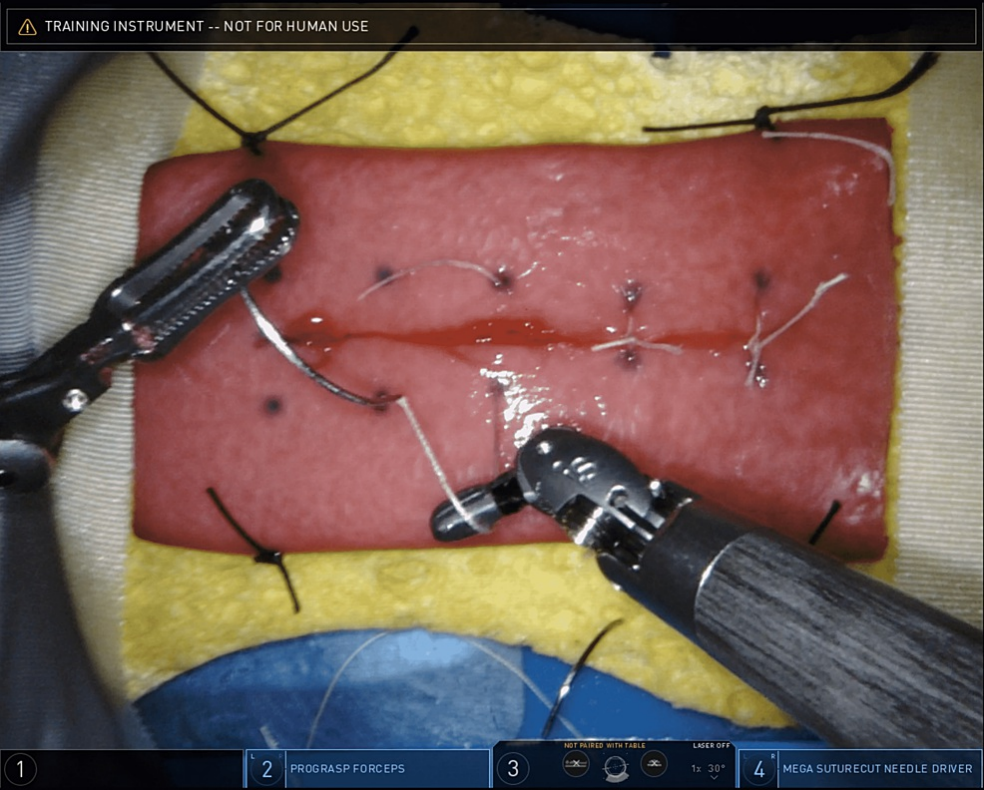
Image of trainee performing the first exercise: Longitudinal enterotomy created with electrocautery followed by simple, interrupted closure using a 3-0 Vicryl suture. Vicryl Suture: Ethicon, Inc., Raritan, New Jersey, United States

Each participant was given two exercises to complete. The start of each exercise involved creating a longitudinal enterotomy using electrocautery with monopolar scissors. In the first exercise, the participants closed the enterotomy with interrupted sutures using the marked dots as landmarks on where to enter and exit the tissue (Figure 3).

**Figure 4:**
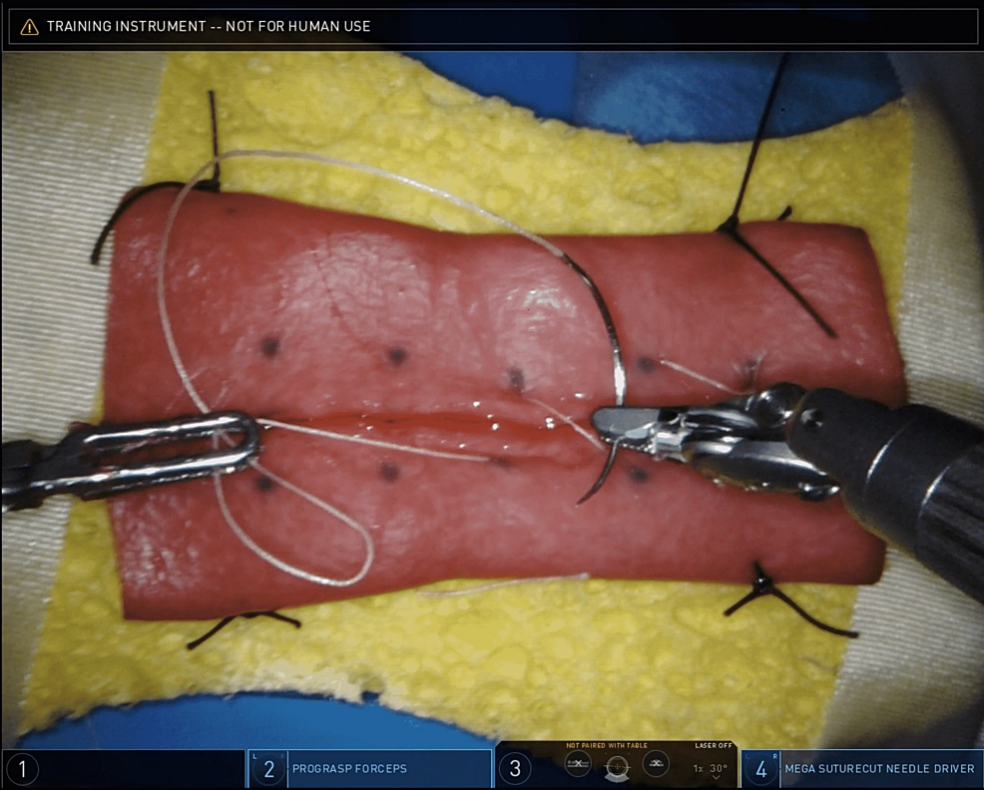
Image of trainee performing the second exercise: Longitudinal enterotomy created with electrocautery followed by running, continuous closure using 3-0 Vicryl suture. Vicryl Suture: Ethicon Inc., Raritan, New Jersey, United States

The bowel tissue was then flipped and resecured to the sponge for the second exercise, during which the participants created an enterotomy in a similar fashion and closed it using a running suture (Figure 4).

General surgery residents in their first and second post-graduate years, as well as third- and fourth-year medical students were invited to participate at two academic institutions. All participation was voluntary. The participants were allotted two 2-0 Vicryl (small half circle (SH) needle) sutures (Ethicon Inc., Raritan, New Jersey, United States) to complete both exercises; one cut to 6 inches (first exercise) and the other to 9 inches (second exercise). There was at least one research staff (general surgery residents trained in robotic surgery) always present to provide bedside support such as switching instruments and providing additional sutures. Participant performance was recorded and graded in real-time for errors defined as instruments out of view, targets missed, torn suture, instrument collisions, air knots, and additional sutures required. Overall feedback was provided to the participant after the completion of the exercises. 

## Discussion

We have described a simple, high-fidelity training platform for cautery. Though there are a variety of curricula for surgical trainees to acquire robotic skills, there are few cost-effective, high-fidelity simulations for basic robotic skills like cautery. While animal and cadaveric training models are still considered the gold standard for simulation training, their cost and ethical implications make them particularly challenging to utilize consistently for basic skills acquisition [10,11]. Virtual reality trainers have proliferated in the field of robotic surgery simulation training; however, they are expensive with initial purchase costs ranging from $45,000 to $125,000 with an additional required annual service contract [12]. They also have been criticized for not adequately simulating electrocautery or providing the same fidelity as live tissue [5,6,13]. Alternatively, inanimate training models provide distinct advantages over those platforms in terms of cost, ethics, and fidelity.

Other inanimate models have been developed for robotic training of procedure-specific skills such as intracorporeal bowel anastomosis and basic surgical skills [14,15]. Some authors argue that dry-lab training on inanimate tissue models could be superior to virtual reality simulation for more advanced skills training [16]. Yet, to our knowledge, there are no cost-effective inanimate models presently available that are capable of simulating electrocautery. Other low-cost inanimate exercises have been published such as the pelvic lymphadenectomy model by Kiely et al. at about $200 [17]. However, this model was not tested with electrocautery and requires five hours of preparation time to create the model. In comparison, our model preparation time was less than 30 minutes on average. 

The training model outlined above accomplishes the goal of teaching a basic robotic skill with high fidelity on a cost-effective platform. Most of the materials used to develop this model are either reusable or easily obtained from a hospital’s sterile processing department. Furthermore, the exercise used along with this model allows participants to apply key elements of robotic surgery: pick and place, two-handed transfer, wrist manipulation, camera control, clutching, suturing, and energy use [18].

Beyond the technical skills developed while performing the exercises, another advantage of this inanimate model is trainee familiarization with the mechanisms of a robotic platform to include driving the tower, docking the arms, camera targeting, and instrument exchange. Most trainees learn these maneuvers via video didactics or from surgical technicians at bedside. In contrast, this training model provided trainees with in-person demonstrations from an experienced user along with hands-on experience setting up the robot. Furthermore, the inanimate training model also allowed the operating learner to actively interact with the support staff, mirroring the relationship between the surgeon, surgical technician, and circulating nurse. This allowed the trainees to practice non-technical skills including closed-loop communication, situational awareness, task management, and teamwork.

A potential limitation of this model is its reliance on fully functional robotic consoles and arms, which in some systems the trainees may have to practice on available robot consoles during non-operative hours, sometimes obligating training during non-business hours. Furthermore, unlike the virtual reality training systems, this training model is best utilized by having an assistant to help with instrument switching, which negates the convenience of solo practice. Technically, the model also does not teach the correct way to close a bowel enterotomy; however, it does effectively teach trainees the previously mentioned skills on life-like tissue in a three-dimensional plane. Better yet, the model is versatile and can be utilized by an advanced user to practice proper enterotomy closure.

## Conclusions

The training exercises and model presented in this paper were designed to allow trainees to improve their basic robotic surgical skills with electrocautery and bowel handling. Materials required for this model can be easily attained, and the set-up can be achieved with basic knowledge of the robot console. Content, face, and construct validity of the model is currently under evaluation.
